# Comparison of HIV-related vulnerabilities between former child soldiers and children never abducted by the LRA in northern Uganda

**DOI:** 10.1186/1752-1505-7-17

**Published:** 2013-08-07

**Authors:** Sheetal Patel, Martin T Schechter, Nelson K Sewankambo, Stella Atim, Charles Oboya, Noah Kiwanuka, Patricia M Spittal

**Affiliations:** 1 School of Population and Public Health, University of British Columbia, 2206 East Mall, Vancouver, BC V6T 1Z3, Canada; 2 Centre for Health Evaluation and Outcome Sciences, Vancouver, BC, Canada; 3 Makerere University College of Health Sciences, Kampala, Uganda; 4 Community-based Researcher, Gulu Town, Uganda; 5 School of Public Health, Makerere University College of Health Sciences, Kampala, Uganda

**Keywords:** Child soldiers, HIV/AIDS, Post-conflict programming, Young people, Northern Uganda

## Abstract

**Background:**

Thousands of former child soldiers who were abducted during the prolonged conflict in northern Uganda have returned to their home communities. Programmes that facilitate their successful reintegration continue to face a number of challenges. Although there is increasing knowledge of the dynamics of HIV infection during conflict, far less is known about its prevalence and implications for population health in the post-conflict period. This study investigated the effects of abduction on the prevalence of HIV and HIV-risk behaviours among young people in Gulu District, northern Uganda. An understanding of abduction experiences and HIV-risk behaviours is vital to both the development of effective reintegration programming for former child soldiers and the design of appropriate HIV prevention interventions for all young people.

**Methods:**

In 2010, we conducted a cross-sectional study of 2 sub-counties in Gulu District. A demographic and behavioural survey was interview-administered to a purposively selected sample of 384 transit camp residents aged 15–29. Biological specimens were collected for HIV rapid testing in the field and confirmatory laboratory testing. Descriptive statistics were used to describe characteristics of abduction. Additionally, a gender-stratified bivariate analysis compared abductees’ and non-abductees’ HIV risk profiles.

**Results:**

Of the 384 participants, 107 (28%) were former child soldiers (61% were young men and 39% were young women). The median age of participants was 20 and median age at abduction was 13. HIV prevalence was similar among former abductees and non-abductees (12% vs. 13%; *p* = 0.824), with no differences observed by gender. With respect to differences in HIV vulnerability, our bivariate analysis identified greater risky sexual behaviours in the past year for former abductees than non-abductees, but there were no differences between the two groups’ survival/livelihood activities and food insufficiency experiences, both overall and by gender. The analysis further revealed that young northern Ugandans in general are in desperate need of education, skills development, and support for victims of sexual violence.

**Conclusions:**

This study persuasively demonstrates that *all* young people in northern Ugandan have been similarly affected by HIV infection during war and displacement. Post-conflict programme planners must therefore abandon rudimentary targeting practices based on abductees as a high-profile category. Instead, they must develop evidence-based HIV interventions that are commensurate with young people’s specific needs. As such programmes will be less stigmatizing, more oriented to self-selection, and more inclusive, they will effectively reach the most vulnerable young people in northern Uganda.

## Background

In northern Uganda, a civil war between the rebel Lord’s Resistance Army (LRA) and the Government of Uganda (GoU) that lasted more than two decades has affected the entire population and its impacts continue to reverberate across generations. Over the course of the conflict, northern Uganda’s civilian population was victim to indiscriminate killings and maimings, widespread forced displacement, and various forms of sexual violence. The social and economic fabrics of their society eroded as children were abducted en masse to become fighters, forced labourers, and sex slaves [1-4]. The data collected by the UNICEF-sponsored Survey of War-Affected Youth (SWAY) in northern Uganda estimated that as many as 66,000 young people between the ages of 14 and 30 were abducted by the LRA during the conflict [5] (a high figure in light of the fact that 250,000 young people are currently involved as child soldiers^a^ in conflicts in 14 countries or territories across the globe [6]). These war-related atrocities and the widespread violation of human rights that accompanied them have left an entire generation of young people traumatized and at a heightened risk of contracting HIV [7,8].

In August of 2006, the GoU and the LRA signed a Cessation of Hostilities Agreement. This significantly improved security for the citizens of northern Uganda and allowed the government to lift restrictions on freedom of movement. As a result, Internally Displaced Persons (IDPs) who were forcibly encamped during the conflict were encouraged to leave primary camp settings and either move back to their ancestral home villages or relocate to transit camps, smaller camps close to villages. By June of 2010, 108 of the 121 officially recognized IDP camps in the Acholi sub-region that was hardest hit by the conflict had been closed. As of December 2010, more than 90 percent of the 1.8 million northern Ugandan IDPs had returned to their home villages, while an estimated 182,000 remained in transit camps [9,10].

During and after the conflict, more than 26,000 formerly abducted individuals have returned home after escaping, being rescued, or being released from duty in the bush [11]. The successful reintegration of these former child soldiers - the social and economic process by which ex-combatants transition into civil society and enter meaningful roles and identities as civilians who are accepted by the public [12] - continues to be a major challenge for northern Ugandan communities. According to Uganda’s Amnesty Commission, only 5,335 out of 26,288 child and youth ex-combatants who renounced and abandoned rebellion had been reintegrated into their communities as of March 2012 (the exercise started in July 2009) [11]. Reintegration programming in northern Uganda has been greatly influenced by fears about the traumatization and dislocation of a ‘lost generation’ of formerly abducted young people. The non-Governmental Organizations (NGOs) that conduct the majority of the reintegration efforts tend to view former abductees strictly in terms of their psychological trauma, and therefore most programmes focus on the provision of broad-based psychological assistance [13]. Viewing former child soldiers through a lens of psychological trauma may cause communities to stigmatize them. This would inhibit communities’ capacity to welcome and accept former child soldiers home, which would in turn compromise the success of the reintegration process.

Ethnographic and quantitative evidence suggests that young ex-combatants are characterized more by resilience than by psychological trauma [5,14-16]. A survey-based assessment of the impacts of abduction on young northern Ugandans suggests that the most pervasive and arguably largest impact of abduction is on education and livelihoods, not physical or psychological trauma [16,17]. Moreover, the survey and interview evidence propose that on average formerly abducted young people appear similar in their mental health to young people in the area who have not been abducted [13], including in assessments based on exposure to traumatic events during the war [18]. Yet, NGO reintegration programmes focused on providing ‘psychosocial care’ to mitigate the psychological trauma among previously abducted child soldiers are available only to that population. Addressing the needs of former abductees is unquestionably important, as they comprise a substantial proportion of the post-conflict population. However, the reintegration programmes exclude thousands of non-abducted individuals who were also deeply affected by the war. Thus, it is necessary to ask: ‘what about the young people who were not abducted but were raped, experienced lack of food or water, suffered ill health without medical care, and endured family separation and/or the death/murder of a family member (all extremely traumatic events)?’

An understanding of abduction experiences is not only key to the development of effective reintegration programming in northern Uganda, but it is also critical to the planning and development of post-conflict programming for non-abductees. The LRA’s recruitment tactics provide a unique opportunity to identify the lasting impacts of abduction. Unlike most other conflict contexts where rebel fighters are a selected segment of the population - including those who volunteer, and those conscripted and screened by army officials - in northern Uganda, LRA recruitment was involuntary and abductions were indiscriminate of all of a young person’s characteristics other than age [13]. Hence, there appear to be no differences in pre-abduction wealth, education, and orphaning between young people in northern Uganda who were abducted and those who were not [13]. Consequently, comparing abductees to non-abductees of the same age and location facilitates an accurate assessment of the long-term impacts of combat, without confounding from pre-existing differences between the two groups. The objective of this study was to determine the effects of abduction and living in the bush on the prevalence of HIV and HIV-risk behaviours among 15-to-29 year olds in Gulu District, northern Uganda. To this end, the analysis included a comparison of ab ductees’ and non-abductees’ HIV risk profiles. The results generated by the comparisons will inform appropriate and effective HIV-responses as well as other post-conflict programming for *all* young people in Gulu District as they continue to transition back to their home villages.

## Methods

### Participants and procedures

The study was conducted between May and December of 2010 in Gulu District, northern Uganda. The two survey areas of the District – Awach and Ongako sub-counties – were chosen randomly from a list of all the sub-counties in Gulu District that had transit camps at the time of data collection (18/23 sub-counties). The study population came from a total of 12 transit camps, which were selected purposively with the guidance of key informants in each sub-county to reflect a suitable range of remoteness/accessibility and resource availability.

#### Sampling

Random and systematic sampling methods were not feasible in the transit camps due to limited data on the population and the unsystematic layouts of the camps. Consequently, a combination of proportional and non-proportional quota sampling methods were employed. The sample size required to estimate a 10% prevalence rate of HIV among young people - with a precision of ±3% (with 95% confidence) - was calculated to be 384. The sample was allocated in proportion to the population size of each sub-county: 216 participants for Ongako sub-county (1.50% of 14,360 people) and 168 for Awach sub-county (1.50% of 11,160). Additionally, we employed a non-proportional quota of 50% male and 50% female. The research team collaborated with hired community mobilizers in each site who assisted with our sampling process and ensured that we met our predetermined sampling quotas.

We calculated that a sub-sample size of 105 former abductees was necessary to have adequate power (>80%) to determine whether there was a meaningful difference in the HIV rates for former abductees and non-abductees. We used a two-tailed two sample test with percentage values to perform this calculation, which included an alpha error level of 5% as well as estimations of sample prevalence based on the informed opinions of the research team (i.e., 17% for abductees and 7% for non-abductees). One hundred and seven of the 384 participants in our study sample self-reported that they had experienced abduction.

#### Interview and HIV testing

A cross-sectional survey design that included the collection of a blood specimen was used to determine HIV prevalence and gather information on socio-demographics, war-related experiences and the sexual behaviours of young people residing in Gulu District transit camps. All participants were interviewed by a well-trained, same-sex Acholi research assistant who was blind to participants’ HIV status and fluent in English as well as the local language of Luo. Following the interview, a trained nurse administered the INSTI HIV-1/HIV-2 Antibody Rapid HIV Test (Biolytical Laboratories). An additional sample of blood was collected from those who tested positive and specimens were transported to the CDC Laboratories at the Uganda Virus Research Institute (UVRI) for confirmatory testing. Serum samples were tested for HIV-specific antibodies using two enzyme-linked immunosorbent assay (ELISA) tests, the Abbott Murex HIV-1/2 ELISA (Murex Biotech Limited, United Kingdom), and the Vironostika HIV Uni-Form II MicroELISA (bioMerieux, Switzerland). In addition, a Western Blot analysis (Calypte Biomedical) was used for definitive characterization when required. Each interview, including specimen collection, took approximately 1.5-2 hours to complete.

#### Ethical considerations

Once heads of household gave us permission to enrol age-eligible household members in our study, the interviewers thoroughly described the research to prospective participants and consent forms were explained in Luo. Written or thumbprint consent was then obtained from all participants. Minors under 18 also required a parent/guardian’s assent unless they were emancipated (married, had children, or were currently pregnant). All interviews and testing took place in a private and quiet place of the participant’s choosing and were conducted anonymously with no names or personal identifiers recorded. We adhered to Ugandan testing guidelines by actively encouraging all participants to receive their test results, but receiving the result was optional and not a requirement for participation. As the interviews and/or HIV testing could precipitate distress, two outreach trauma counsellors travelled with the research team to the field daily in order to ensure that any participant requesting psychosocial support received it immediately. In addition, referrals were made available for follow-up HIV care. As involvement in the study took participants away from their gardens and household activities, each respondent received 4000 Ugandan Shillings (~$2USD) as compensation.

Ethical approval for this study was obtained from the University of British Columbia Providence Healthcare Research Ethics Board, Vancouver, Canada; the Research and Ethics Committee for the Child Health and Development Centre, Makerere University, College of Health Sciences, Kampala, Uganda; the Uganda National Council of Science and Technology, and; the Republic of Uganda, Office of the President.

### Statistical analysis

A descriptive/bivariate analysis without any regression modelling was deemed appropriate for fulfilling the study’s objective to determine the effect that abduction and living in the bush as a child soldier had on HIV-prevalence and risk behaviours. All analyses were conducted using SPSS Version 19.0. Point estimates of HIV prevalence and corresponding 95% confidence intervals were calculated separately for all participants; participants who had and had not been abducted by the LRA, and; female and male abductees. Descriptive statistics were used to describe the characteristics of abduction for all abductees as well as male and female abductees. A series of bivariate analyses were then conducted (first among all participants and then stratified by gender) to examine how abductees and non-abductees differed with respect to socio-demographic characteristics; war-related experiences; sexual behaviours; survival/livelihood circumstances; HIV/AIDS prevention practices, and; health status. Categorical variables were compared using Pearson’s chi-square and when 25% or more of the expected cell frequencies in a contingency table were less than 5, Fisher’s exact test was used. For continuous variables, the Wilcoxon rank sum test compared medians of non-normally distributed factors. All reported p-values are two-sided.

## Results

### Sample characteristics

Characteristics of the sample are provided in Table 1. An equal proportion of young women (50%) and young men (50%) were included in the sample and 107 (28%) respondents self-reported having experienced abduction. The median age of respondents was 20 years and the main ethnic group was Acholi (98%). Nearly 40% of participants were married and 25% were in school. Over half (58%) of the respondents had been displaced for more than 10 years, and 68% had been living in a transit camp for 1–5 years.

**Table 1:** Sample characteristics of participants (N = 384)

**Characteristics**	**Number (%)**
Female	192 (50.0)
Age, yr, median (range)	20 (15-29)
Abducted by the LRA	107 (27.9)
**Sub-county**	
Awach	168 (43.8)
Ongako	216 (56.2)
**Religion**	
Roman Catholic	311 (81.0)
Protestant Church of Uganda	47 (12.2)
Muslim	14 (3.7)
Pentecostal Christian	12 (3.1)
**Ethnicity**	
Acholi	376 (97.9)
Other	8 (2.1)
**Marital status**	
Currently married	150 (39.0)
Other	82 (21.4)
Never married	152 (39.6)
**School status**	
Ever school	374 (97.4)
In school	96 (25.0)
Dropped out	278 (72.4)
**Personal income based on previous month**	
<25,000 UGS^*1^	232 (60.4)
25,000-50,000 UGS	60 (15.6)
>50,000 UGS	57 (14.8)
Dependent on parents	26 (6.8)
**Duration of displacement in camps**	
Never	12 (3.1)
3–5 years	28 (7.3)
5–10 years	85 (22.1)
More than 10 years	221 (57.6)
Since I was born	38 (9.9)
**Duration of stay in transit camp**	
1–12 months	85 (22.1)
13 months- 2 years	166 (43.2)
3–5 years	97 (25.3)
More than 5 years	36 (9.4)

^*1^Approximately $10USD.

### Characteristics of abduction

Characteristics of abduction are presented in Table 2. Out of the 107 (28%) respondents who reported that they had been abducted by the LRA, 42 (39%) were young women and 65 (61%) were young men. The median age at abduction was 13years (12 years among females and 14 years among males). Sixty-seven percent of former child soldiers reported that they were abducted from the IDP camp where they lived and 12% indicated that they had been abducted from a school building or school grounds (data not shown). Length of abduction ranged from one week to 15 years; 56% of the abductees had been held captive for at least four months, 35% were held for a year or more, and only 4% were held for more than five years. Median length of time in captivity was 14 months and the median number of years since returning from the bush was 7.5, with males reporting a longer duration of abduction and correspondingly shorter length of time as a returnee.

**Table 2:** Characteristics of formerly abducted participants

	**Total (N=107)**	**Females (N=42)**	**Males (N=65)**	
**Variable**	**N (%)**	**N (%)**	**N (%)**	**p value**
Age of abduction, yr, median (range)	13 (6–21)	12 (6–19)	14 (7–21)	**0.019**
Length of time in captivity, mnths, median (range)	4 (0.25-180)	3 (0.25-180)	4 (0.25-99)	0.870
Length of time since return from bush, yrs, median (range)	7.5 (0.08-19)	10 (0.08-17)	6 (0.08-19)	**0.001**
**While in the bush:**				
stayed in difficult circumstances	101 (94.4)	37 (88.1)	64 (98.5)	**0.033**
made to carry heavy loads	100 (93.5)	41 (97.6)	59 (90.8)	0.242
forced into military training	61 (57.0)	17 (40.5)	44 (67.7)	**0.014**
made to fight	53 (49.5)	13 (31.0)	40 (61.5)	**0.005**
personally kill another person	35 (32.7)	14 (33.3)	21 (32.3)	0.912
witness someone being killed	72 (67.3)	29 (69.0)	43 (66.2)	0.755
made to abduct other children	71 (66.4)	20 (47.6)	51 (78.5)	**0.001**
looted properties and burned houses	75 (70.1)	22 (52.4)	53 (81.5)	**0.001**
was injured	78 (72.9)	33 (78.6)	45 (69.2)	0.288
was seriously beaten	82 (76.6)	31 (73.8)	51 (78.5)	0.579
was sexually abused	21 (19.6)	14 (33.3)	7 (10.8)	**0.004**
had access to condoms	4 (3.7)	3 (7.1)	1 (1.5)	0.297
gave birth	*-----*	5 (11.9)	*-----*	*-----*
given as a wife	*-----*	17 (40.5)	*-----*	*-----*
# of times given as a wife, median (range)	*-----*	1 (1–3)	*-----*	*-----*
given a wife	*-----*	*-----*	11 (16.9)	*-----*
# of times given a wife, median (range)	*-----*	*-----*	1 (1–2)	*-----*

The vast majority of the formerly abducted participants (94%) reported that living in the bush as an LRA captive was difficult and that they were made to carry heavy loads (of water, arms and other supplies). More than half of the abductees (57%) were forced into military training and half (50%) were made to fight. A third (33%) reported that they had personally killed another person, while over two-thirds (67%) had witnessed someone being killed. Sixty-six percent were made to abduct other children and 70% were required to loot civilians’ properties and burn civilians’ houses. Nearly three quarters (73%) were injured while in captivity and 77% were severely beaten. One-fifth (20%) were sexually abused and only 4% had access to condoms during their abduction. Over 40% of female abductees reported that they had been given to a man as his wife and 12% had given birth while living in the bush; the median number of times given as a wife was 1. In contrast, 17% of the formerly abducted young men had been given a wife while in captivity; the median number of times they were given a wife was 1.

There were other significant differences between the abduction experiences of young women and young men. A significantly higher proportion of young men reported that they had looted properties and burned houses (82% vs. 52%, *p* = 0.001); been made to abduct other children (79% vs. 48%, *p* = 0.001); been forced into military training (68% vs. 41%, *p* = 0.014), and; been made to fight (62% vs. 31%, *p* = 0.005). It is not surprising that young women were significantly more likely to report being sexually abused (33% vs. 11%, *p* = 0.004) however, it is interesting to note that no significant difference was found in the proportions of young women and young men who had personally killed someone (33% vs. 32%, *p* = 0.912).

### Prevalence of HIV

As shown in Table 3, 49 of the total 384 participants sampled tested positive for the HIV antibody, yielding an overall prevalence rate of 12.8% (95% CI: 9.6%, 16.5%). HIV prevalence was 12.1% (95% CI: 6.6%, 19.9%) for formerly abducted participants, compared to 13.0% (95% CI: 9.3%, 17.5%) for participants who had never experienced abduction. Eight (12.3%; 95% CI: 5.4%, 22.8%) formerly abducted male participants were HIV-positive compared to 11 (8.7%; 95% CI: 4.4%, 14.9%) male participants who had not been abducted. Five (11.9%; 95% CI: 3.9%, 25.6%) formerly abducted female participants were HIV-positive compared to 25 (16.7%; 95% CI: 11.1%, 23.6%) female participants who had not been abducted. No significant differences in HIV prevalence were demonstrated between formerly abducted and non-abducted participants overall (*p* = 0.824) or by gender (Males: *p* = 0.423; Females: *p* = 0.453).

**Table 3:** Prevalence of HIV infection among study participants who were abducted and those who were not

**All participants**			
**prevalence estimate (%)**			
**[95% CI]**			
***(# Infected/Total N)***			
12.8			
[9.6-16.5]			
*(49/384)*			
	**Formerly abducted**	**Not abducted**	
	**Prevalence estimate (%) [95% CI]**	**Prevalence estimate (%) [95% CI]**	**p value**
**Group**	***(# Infected/Total N)***	***(# Infected/Total N)***	
All participants	12.1	13.0	
	[6.6-19.9]	[9.3-17.5]	0.824
	*(13/107)*	*(36/277)*	
Males	12.3	8.7	
	[5.4-22.8]	[4.4-14.9]	0.423
	*(8/65)*	*(11/127)*	
Females	11.9	16.7	
	[3.9-25.6]	[11.1-23.6]	0.453
	*(5/42)*	*(25/150)*	

### Comparisons among abducted and non-abducted participants

All gender-stratified bivariate comparisons between participants who had been abducted (n = 107) and those who had not been abducted (n = 277) are summarized in Table 4 and are grouped into six categories of interest.

**Table 4:** Comparison of socio-demographic and behavioural characteristics of formerly abducted and non-abducted participants stratified by gender

	**Male**			**Female**			**Total**		
	**Formerly abducted (N=65)**	**Not abducted (N=127)**		**Formerly abducted (N=42)**	**Not abducted (N=150)**		**Formerly abducted (N=107)**	**Not abducted (N=277)**	
**Variable**	**N (%)**	**N (%)**	**p value**	**N (%)**	**N (%)**	**p value**	**N (%)**	**N (%)**	**p value**
***Demographics*** &***Background Information***									
Age, yr, median (range)	22 (15–29)	19 (15–29)	**0.015**	23 (15–29)	19 (15–29)	**0.004**	23 (15–29)	19 (15–29)	**<0.001**
Awach sub-county	35 (53.8)	49 (38.6)	**0.044**	19 (45.2)	65 (43.3)	0.826	54 (50.5)	114 (41.2)	0.099
Ever married	40 (61.5)	52 (40.9)	**0.007**	36 (85.7)	104 (69.3)	**0.035**	76 (71.0)	156 (56.3)	**0.008**
Age at 1^st^ marriage, yr, median (range)	18 (12–24)	18 (12–25)	0.099	15 (9–22)	16 (13–22)	**0.002**	16 (9–24)	17 (12–25)	0.118
In school	13 (20.0)	43 (33.9)	**0.046**	3 (7.1)	37 (24.7)	**0.013**	16 (15.0)	80 (28.9)	**0.005**
>10 yrs IDP camp living	39 (60.0)	68 (53.5)	0.394	31 (73.8)	121 (80.7)	0.333	70 (65.4)	189 (68.2)	0.598
Living with entire family	11 (16.9)	24 (18.9)	0.514	14 (33.3)	47 (31.3)	0.598	25 (23.4)	71 (25.6)	0.800
Night commuter^*1^	43 (66.2)	105 (82.7)	**0.010**	33 (78.6)	97 (64.7)	0.088	76 (71.0)	202 (72.9)	0.709
Monthly income < 25,000 UGS^*2^	34 (52.3)	66 (52.0)	0.685	33 (78.6)	99 (66.0)	0.117	67 (62.6)	165 (59.6)	0.380
***Sexual Activity/Practices*** &***Relationships***									
Sexually active	55 (84.6)	90 (70.9)	**0.036**	40 (95.2)	116 (77.3)	**0.009**	95 (88.8)	206 (74.4)	**0.002**
Age at 1st sex, yr, median (range)	17 (9–24)	17 (8–25)	0.995	15 (6–18)	16 (8–22)	**0.009**	16 (6–24)	16 (8–25)	0.224
Non-consensual 1^st^ sex^a^	9 (16.4)	2 (2.2)	**0.001**	20 (50.0)	21 (18.1)	**<0.001**	29 (30.5)	23 (11.2)	**<0.001**
1st sex partner ≥10 yrs older^a^	4 (7.3)	2 (2.2)	**0.008**	21 (52.5)	24 (20.7)	**<0.001**	25 (26.3)	26 (12.6)	**<0.001**
Occupation of 1^st^ sex partner military/rebel^a^	7 (12.7)	0 (0)	-----	17 (42.5)	3 (2.6)	**<0.001**	24 (25.3)	3 (1.5)	**<0.001**
Had sex past 12 months	47 (85.5)	72 (80.0)	0.406	24 (60.0)	91 (78.4)	**0.022**	71 (74.7)	163 (79.1)	0.480
Had sex past 6 months	44 (80.0)	71 (78.9)	0.873	20 (50.0)	72 (62.1)	0.250	64 (67.3)	143 (69.4)	0.819
Number of sex partners past 6 months, median (range)^b^	2 (1–9)	1 (1–5)	**0.055**	1 (1–2)	1 (1–2)	0.621	2 (1–9)	1 (1–5)	**0.021**
Ever practice dry sex^*3,a^	35 (63.6)	40 (44.4)	**0.038**	23 (57.5)	54 (46.6)	0.312	58 (61.1)	94 (45.6)	**0.018**
Currently have sex partner besides your spouse^c^	6 (19.4)	8 (47.1)	0.063	2 (10.0)	1 (1.2)	**0.037**	8 (15.7)	9 (9.1)	0.227
***Gender-based Violence***									
Ever experience physical/sexual/verbal abuse from sexual partner^a^	28 (50.9)	26 (28.9)	**0.001**	32 (80.0)	64 (55.2)	**<0.001**	60 (63.2)	90 (43.7)	**<0.001**
Experience physical/sexual/verbal abuse past 12 months^d^	16 (34.0)	14 (19.4)	**0.014**	11 (45.8)	26 (28.6)	**0.050**	27 (38.0)	40 (24.5)	**0.012**
Ever been raped^a^	11 (20.0)	3 (3.3)	**0.001**	21 (52.5)	24 (20.7)	**<0.001**	32 (33.7)	27 (13.1)	**<0.001**
Age at rape, yr, median (range)	17 (12–24)	16 (11–19)	0.607	14 (6–19)	14 (8–23)	0.595	14 (6–24)	14 (8–23)	0.992
Perpetrator ≥ 10 yrs older	4 (36.4)	3 (100.0)	0.230	15 (71.4)	13 (54.2)	0.378	19 (59.4)	16 (59.3)	1.000
Ever beat sexual partner^a^	13 (23.6)	22 (24.4)	1.000	*-----*	*-----*	*-----*	13 (13.7)	22 (10.7)	0.566
***Survival/Livelihood Activities &Food Security***									
Main means of livelihood –Limited cultivation	51 (78.5)	99 (78.0)	0.875	30 (71.4)	91 (60.7)	0.150	81 (75.7)	190 (68.6)	0.242
Main means of livelihood –Brew alcohol	*-----*	*-----*	*-----*	1 (2.4)	11 (7.3)	0.432	1 (.9)	11 (4.0)	0.216
Ever survival sex work^*4,a^	*-----*	*-----*	*-----*	2 (5.0)	9 (7.8)	0.860	2 (2.1)	9 (4.4)	0.539
Current lack of food and/or water	17 (26.2)	26 (20.5)	0.372	26 (61.9)	84 (56.0)	0.494	43 (40.2)	110 (39.7)	0.932
Gone hungry past 12 months	28 (43.1)	61 (48.0)	0.515	27 (64.3)	91 (59.0)	0.670	55 (51.4)	152 (54.9)	0.541
***HIV/AIDS Prevention*** &***Condom Use***									
Ever used a condom	49 (75.4)	79 (62.2)	0.067	19 (45.2)	61 (40.7)	0.595	68 (63.6)	140 (50.5)	**0.022**
Condom use past 6 months^b^	20 (45.5)	31 (43.7)	1.000	6 (30.0)	21 (29.2)	1.000	26 (40.6)	56 (39.2)	0.878
Condom use last sex^a^	23 (41.8)	32 (35.6)	0.451	8 (20.0)	23 (19.8)	0.981	31 (32.6)	55 (26.7)	0.290
Consistent condom use	17 (34.7)	24 (30.4)	0.769	1 (5.3)	14 (23.0)	0.085	18 (26.5)	38 (27.1)	0.903
Ever HIV test	55 (84.6)	88 (69.3)	**0.021**	39 (92.9)	132 (88.0)	0.576	94 (87.9)	220 (79.4)	0.071
No. of HIV tests, median (range)	11 (0–27)	10 (0–30)	0.367	15 (0–23)	15 (0–25)	0.529	13 (0–27)	15 (0–30)	0.577
Know any sexual partner’s HIV status^a^	43 (78.2)	84 (93.3)	**0.017**	29 (72.5)	86 (74.1)	0.991	62 (65.3)	180 (87.4)	**<0.001**
***Health Status*** &***Service Utilization***									
Never sought healthcare outside of home	19 (29.2)	46 (36.2)	0.138	9 (21.4)	52 (34.7)	0.148	28 (26.2)	98 (35.4)	0.085
Had a health problem past 12 months	31 (47.7)	49 (38.6)	0.226	33 (78.6)	110 (73.3)	0.491	64 (59.8)	159 (57.4)	0.668
Experienced ill health without medical care past 3 months	13 (20.0)	13 (10.2)	0.104	8 (19.0)	9 (6.0)	**0.014**	21 (19.6)	22 (7.9)	**0.001**
Very likely to have been exposed to HIV	9 (13.8)	5 (3.9)	0.091	7 (16.7)	28 (18.7)	0.631	16 (15.0)	33 (11.9)	0.631
Ever consumed alcohol	18 (27.7)	33 (26.0)	0.800	2 (4.8)	5 (3.3)	0.649	20 (18.7)	38 (13.7)	0.222
Ever had STI besides HIV^a^	9 (16.4)	9 (10.0)	0.384	6 (15.0)	11 (9.5)	0.489	15 (15.8)	20 (9.7)	0.185
Had any STI symptoms past 12 months^d^	3 (6.4)	6 (8.3)	1.000	21 (87.5)	33 (36.3)	**<0.001**	24 (33.8)	39 (23.9)	0.149
Ever pregnant^a^	*-----*	*-----*	*-----*	36 (90.0)	99 (85.3)	0.654	*-----*	*-----*	*-----*
Age at 1st pregnancy, yr, median (range)	*-----*	*-----*	*-----*	16 (10–20)	17 (13–22)	0.105	*-----*	*-----*	*-----*
Ever circumcised	4 (6.2)	7 (5.5)	1.000	*-----*	*-----*	*-----*	*-----*	*-----*	*-----*
Age of circumcision, yr, median (range)	13 (0–18)	12 (0–24)	0.847	*-----*	*-----*	*-----*	*-----*	*-----*	*-----*

^*1^Leaving your family hut at night to sleep elsewhere due to security and privacy concerns.

^*2^Approximately $10USD.

^*3^Sexual intercourse without foreplay or lubrication so that the vagina is dry upon penetration.

^*4^Exchanging sex for food, shelter, money, gifts.

^a^Among those reporting ever having had sex, Abducted males (*n*=55) Non-abducted males (*n*=90); Abducted females (*n*=40) Non-abducted females (*n*=116).

^b^Among those reporting sexual activity in previous 6 months, Abducted males (*n*=44) Non-abducted males (*n*=71); Abducted females (*n*=20) Non-abducted females (*n*=72).

^c^Among those currently married, Abducted males (*n*=31) Non-abducted males (*n*=17); Abducted females (*n*=20) Non-abducted females (*n*=82).

^d^Among those reporting sexual activity in previous 12 months, Abducted males (*n*=47) Non-abducted males (*n*=72); Abducted females (*n*=24) Non-abducted females (*n*=91).

#### Demographics and background information

Respondents who self-reported that they had experienced abduction were older (median age of 23 vs. 19 years, *p* < 0.001), more likely to have ever been married (71% vs. 56%, *p* = 0.008), and less likely to be in school (15% vs. 29%, *p* = 0.005) than non-previously abducted participants. Among male participants, a significantly higher proportion of former abductees than non-abductees were residents of Awach sub-county (54% vs. 39%, *p* = 0.044). In addition, a significantly lower proportion of former male abductees than non-abductees reported that they had night-commuted (leaving one’s family hut to sleep elsewhere for reasons of security and privacy) during the war (66% vs. 83%, *p* = 0.010). Among females, former abductees reported a significantly lower median age at first marriage than non-abductees (15 vs. 16 years, *p* = 0.002). Similarly high proportions of abductees and non-abductees earned a monthly income of less than 25,000 Ugandan Shillings (UGS), approximately $10USD (63% vs. 60%, *p =* 0.380).

#### Sexual activity and practices

Eighty-nine percent of former abductees had had sex before, in comparison to 74% of non-abductees (*p* = 0.002) and this significantly higher proportion applied to both male and female abductees. A significantly greater proportion of former abductees than non-abductees reported that their sexual debut was non-consensual (31% vs. 11%, *p* < 0.001); that their first sex partner was 10 or more years older than they (26% vs. 13%, *p* < 0.001), and; that their first sex partner was a military/rebel soldier (25% vs. 2%, *p* < 0.001). All three of these significant differences were also demonstrated separately for male and female participants. Moreover, among female participants alone, former abductees reported a significantly lower median age at sexual debut (15 vs. 16 years, *p* = 0.009) than non-abductees.

Similar proportions of sexually active former abductees and non-abductees reported that they had had sex in the previous 6 months (67% vs. 69%, *p* = 0.819) and these proportions did not differ significantly by gender. Former abductees reported a greater median number of sex partners in the previous 6 months (2 vs. 1, *p* = 0.021) and this significant difference was also demonstrated between former male abductees and male non-abductees (2 vs. 1, *p* = 0.055). A significantly greater proportion of formerly abducted participants reported that they had practiced dry sex (61% vs. 46%, *p* = 0.018) and again, this difference was also demonstrated between male abductees and non-abductees (64% vs. 44%, *p* = 0.038). Among females, former abductees were more likely than non-abductees to indicate that they currently had a sex partner besides their spouse (10% vs. 1.2%, *p* = 0.037).

#### Gender-based violence

A significantly greater proportion of former abductees than non-abductees reported that they had been physically/sexually/verbally abused by any of their sexual partners (63% vs. 44%, *p* < 0.001); this was also the case for participants who had experienced abuse in the previous 12 months (38% vs. 25%, *p* = 0.012). These significant differences were also demonstrated separately for male and female participants. Similarly, significantly greater proportions of former abductees versus non-abductees indicated that they had been raped, both overall (34% vs. 13%, *p* < 0.001) as well as separately by gender (Males: 20% vs. 3%, *p* = 0.001; Females: 53% vs. 21%, *p* < 0.001). A high proportion of former abductees and non-abductees who had been raped reported that their perpetrator was 10 or more years older than them (59% overall). Finally, nearly 25% of sexually experienced young men reported that they had beaten their partner on at least one occasion, with no demonstrated difference between former abductees and non-abductees.

#### Survival/livelihood activities and food security

No significant differences were found in the survival/livelihood activities and food insufficiency experiences of former abductees and non-abductees, both overall and by gender. Limited agricultural cultivation was a primary means of livelihood for similar proportions of former abductees and non-abductees overall (*p* = 0.242), as well as separately by gender (Males: *p* = 0.875; Females: *p* = 0.150). Only female participants reported that they brewed alcohol as their primary means of livelihood, with no significant difference demonstrated between female abductees and non-abductees (*p* = 0.432). Similarly, the 11 participants who reported that they had exchanged sex for food, shelter, money or gifts (i.e., survival sex) were also all female, with no significant difference between female abductees and non-abductees (*p* = 0.860). Comparable proportions of former abductees and non-abductees experienced food insufficiency; 40% of both groups were currently experiencing a lack of food and/or water (*p* = 0.932), while 51% of former abductees and 55% of non-abductees indicated that they had gone hungry in the previous 12 months (*p* = 0.541).

#### HIV/AIDS prevention and condom use

A significantly greater proportion of formerly abducted participants than non-abducted participants reported that they had ever used a condom (64% vs. 51%, *p* = 0.022), although there was no significant difference in the two groups with respect to condom use in the previous 6 months, condom use during last sexual experience, or consistent condom use. In general, only 19% of the sexually experienced young people in this study indicated that they used condoms consistently. The vast majority of both former abductees (88%) and non-abductees (79%) had had at least one previous HIV test, while a significantly greater proportion of male abductees had ever had a HIV test than male non-abductees (85% vs. 69%, *p* = 0.021). Former abductees had taken a lower median number of HIV tests in their lifetime than non-abductees, but this difference was not significant (13 vs. 15 times, *p* = 0.577). A significantly smaller proportion of formerly abducted participants than non-abducted participants had knowledge of their partner’s HIV status (65% vs. 87%, *p* < 0.001). This significant difference was also demonstrated between male abductees and male non-abductees (78% vs. 93%, *p* = 0.017).

#### Health status and service utilization

One-third of all participants reported that they had never sought healthcare outside of their home and there were no significant differences between abductees and non-abductees. Similarly, 58% of all respondents indicated that they had had a health problem in the previous 12 months (73% reported the health problem to be malaria) and there were no significant differences between abductees and non-abductees. In contrast, a significantly higher proportion of formerly abducted participants than non-abducted participants had experienced ill health without being medically treated for it in the previous 3 months (20% vs. 8%, *p* = 0.001); this significant difference was also demonstrated between female abductees and female non-abductees (19% vs. 6%, *p* = 0.014). No significant differences were identified between the proportions of former abductees and non-abductees who perceived that they had very likely been exposed to HIV; who reported having ever consumed alcohol, and; who indicated ever having had an STI other than HIV. Among the young women who had had sex in the previous 12 months, a significantly higher proportion of former abductees than non-abductees indicated that they had had STI symptoms in the same time period (88% vs. 36%, *p* < 0.001). Eighty-seven percent of sexually active females reported past pregnancies and there was no significant difference in the proportions of ‘ever pregnant’ between female abductees and non-abductees (90% vs. 85%, *p* = 0.654). Six percent of young men reported that they had been circumcised, with no significant difference between male abductees and non-abductees (*p* = 1.000).

## Discussion

This study investigated the effect of abduction on HIV prevalence and risk behaviours among former abductees in Gulu District, northern Uganda. Therein, it compared formerly abducted participants’ HIV risk profiles with those of individuals who had not been abducted. This study provides evidence that young people who have not experienced abduction are just as likely to contract HIV infection as those who have been abducted (13% vs. 12%, *p* = 0.824). This is contrary to what was initially hypothesized and what NGO reports and media stories would lead readers to believe. Although further research is recommended to better understand the similarity in HIV rates for these two groups of young people, one potential explanation is presented below. Previous research on the experiences of former female child soldiers in northern Uganda [19-22] concurs that girls who were abducted were not violated on their way to the LRA stronghold in Sudan. If a girl was raped, her perpetrator was killed. Upon reaching the Sudan, the girls were delegated to the highest-ranking men. The onset of menstruation was the sign of maturity for Acholi girls and abducted young girls who had had their first menstruation immediately became ‘wives’. Pre-menstrual girls were kept as house helpers (‘*ting-ting’*) until they started menstruating*.* A girl’s commander assigned her a ‘husband’ and she had no say in the matter. If a girl were found having sex with a man other than her ‘husband’ , she would be court-martialled and executed by a firing squad. Girls were required to remain with their husbands until they died. When a husband did die, men in positions of higher command decided where his ‘wife’ went next. The abducted girls described these experiences as ‘sexual slavery’ because there was no love, no consent and no bride wealth paid to their parents [22].

A report by the SWAY research programme (2008) points out that female abductees also performed vital combat and support roles within the LRA and suggests that most were not used primarily as ‘sexual slaves’ [23]. At the same time, Park (2006) notes from research conducted in Sierra Leone that many captive wives described their forced marriage as a protective factor while in the bush, as their ‘husband’ gave them food and protected them from being sexually abused by multiple men [24]. Nonetheless, many formerly abducted young women are stigmatized by their communities after returning from the bush because of condemnatory perceptions of their previous sexual enslavement. Based on initial reports by the media and by NGOs on female returnees in northern Uganda it was believed that they had been shared indiscriminately amongst rebel commanders as sexual “free for alls” [20,22]. This perception persisted despite the fact that captive menstruating girls were given only to *one* man within the confines of a ‘forced marriage’ and rape by someone other than their husband was extremely rare. The median number of times that female abductees in this study reported being given as a wife was one and the median number of times that male abductees were given a wife was also one. Hence, a girl who was never abducted may have had more sexual partners than an abducted girl who had lived in the bush. This information illustrates that conflict-affected abductees and non-abductees may experience vulnerability to HIV infection in different ways than may be originally understood (i.e., different exposure opportunities). However, their overall susceptibility to HIV infection remains analogous, as was demonstrated in this study in finding similar rates of infection among abductees and non-abductees (12% vs. 13%, *p* = 0.824). Our finding suggests that both groups have been deeply and equally impacted by HIV infection during the war and thus both deserve commensurate attention and access to appropriate HIV-related programming during the post-conflict period.

The study did produce some evidence to support its secondary hypothesis that young people who have experienced abduction and lived in the bush will exhibit more HIV-risk behaviours than young people who were not abducted. Specifically, we found that participants who had been abducted were more likely to report a greater median number of sex partners in the previous 6 months than those who had not been abducted and were also less likely to have knowledge of their sexual partner’s HIV status. Our comparison of former male abductees and male non-abductees also demonstrated both of these significant associations. Furthermore, comparisons among female participants revealed that, former abductees were more likely than non-abductees to report currently having a sex partner other than their spouse and having had STI symptoms in the previous 12 months. In fact, 88% of formerly abducted females who had been sexually active in the past 12 months also experienced STI symptoms in the same time period (in comparison to 36% of non-abducted females, *p* < 0.001). All of these significant findings illustrate that HIV-related risk behaviours were elevated among formerly abducted young people within the previous 12 months and it is likely that this population’s risk of HIV remains high.

Various reports on former child soldiers in northern Uganda indicate that the LRA rebels were reticent to abduct older children because of the possibility that these children might already be infected with HIV [20,25]. It is fair to assume then that young people were likely to be HIV-negative when they were abducted. Given that sexual partners were limited during captivity, it is probable that many former abductees who tested positive for HIV in this study were still HIV-negative when they left the bush, but contracted the virus after returning home. The literature does suggest that experiencing trauma in childhood may increase the likelihood of subsequently engaging in high-risk sexual behaviour, thus increasing the risk of contracting HIV or other STIs [26-28]. Our study findings corroborate this literature in finding that former abductees compared to non-abductees exhibited greater HIV-related sexual behaviours in the previous 12 months. At the same time, however, it is not clear why abductees’ greater risk behaviour in the past year is not reflected in a higher prevalence of HIV infection compared to non-abductees in our sample. One potential explanation may be that what are thought to be risk factors for HIV infection are not actually so. Another possibility is that abductees’ experiences in the bush have caused them to adopt behaviours that lower their risk of contracting HIV. For example, a greater proportion of abductees compared to non-abductees reported having at some point used a condom (64% vs. 51%, *p* = 0.022), and believed that using condoms most effectively prevented infection (36% vs. 23%, *p* = 0.014). Abductees’ affinity to use a condom may be increased after they return from the bush where their access to condoms was basically non-existent, and a substantial amount of sexual health care is offered to them as they transition back to their communities, further enabling this protective behaviour. Nonetheless, further in-depth research is needed to better understand the experiences and risk environments for abductees both during and after captivity.

Assistance targeted to formerly abducted young people in northern Uganda has been provided in two main forms: reinsertion assistance and longer-term reintegration and development services. The primary instruments of the former have been the provision of interim care at reception centres, Amnesty Certificates, and reinsertion packages to former abductees who report to the centres or to the Amnesty Commission [13]. The focus of the reception process and interim care has included access to basic health services; basic counselling; family tracing and reunification, and; broad-based measures aimed to sensitize communities to former abductees’ needs. Most former abductees stay at the reception centres for two to six weeks. The centres offer very limited follow-up care and only occasional assistance for education, health, vocational training, food, or shelter [13] – despite evidence that former child soldiers desperately need education and a means of earning a livelihood [16,17]. Forcible recruitment creates a need for education because abductees do not attend school when they are living with the rebel group in the bush. This impact is obviously greatest for long-term abductees who on average attain one to two fewer grades and are more than twice as likely to be illiterate than young people who were not abducted [16]. It is therefore not surprising that former male abductees in northern Uganda were less than half as likely to be skilled workers or businessmen than their non-abducted male peers, and hence earned 33% less than them in daily wages [17]. This was not only because they had lost time at school, but also because of the lingering effects of serious injuries incurred during the war. The education of women who returned from the LRA with children is even more severely affected. Unlike other abductees, these young mothers almost never go back to school, largely because of their childcare responsibilities [20,23]. Our research corroborates the results of these studies in finding that former abductees were significantly less likely to be in school than non-abductees and that this disparity was most pronounced among female participants (non-abducted young women were three times more likely to be in school than young women who had been abducted).

Both traditional and alternative forms of education broadly support the reintegration of former child soldiers in a number of ways. For example, Machel (1996) emphasizes that literacy and skills learning are essential to the economic security of former child soldiers and often determine whether or not they successfully reintegrate into their communities [29]. Perhaps more importantly, Betancourt et al. (2008) points out that returning to school can help former abductees begin to identify as someone other than a soldier or a victim [30]. In other words, educational programmes may help to ‘normalize’ life for returning child soldiers and allow them to develop an identity and sense of self-worth that are not associated with being a soldier. Furthermore, Betancourt et al. (2008) suggest that the true reintegration of former child soldiers depends on access to educational and training opportunities that will support them to achieve greater self-sufficiency and increased productivity within their communities [30]. The Ugandan government and NGOs have mainly focused their educational efforts on the primary school system and vocational training programmes. Unfortunately, best practices programmes that provide secondary school scholarships, accelerated adult education, and child-care and meals at school for the children of students are extremely rare and serve only a small fraction of the under-educated population that includes former abductees [16]. Our findings reiterate the importance of providing former child soldiers with appropriate educational opportunities, but also indicate that there is an urgent need for best practices programmes to be expanded and made accessible to non-abductees as well as abductees. The fact that 74% of the 374 participants in this study who had attended school at some point had subsequently dropped out with no hope of re-joining provides evidence of this claim. A lack of money for fees and school materials (i.e., uniforms, books, pencils), feeling too old to resume schooling after being away from it for a substantial time, and pregnancy were the main reasons given by participants for dropping out of school. Accordingly, any educational programmes for former abductees in northern Uganda must be introduced in tandem with larger educational support programmes that include *all* war-affected young people.

The other primary instruments of reinsertion assistance offered to former abductees in northern Uganda are Amnesty Certificates and reinsertion packages. Since 2000, government legislation has granted blanket amnesty to members of armed groups who have renounced and abandoned rebellion [11]. Eligibility criteria for an Amnesty Certificate stipulate that bearers must be Ugandan citizens, be 12 years or older at time of return, and have fought against the Government of Uganda. The Amnesty Commission, since 2006, has retroactively paid out reinsertion packages to former abductees who are holders of an Amnesty Certificate [11]. The package intends to help former abductees rebuild their lives by providing them with household items (a mattress, blanket, cups and plates), agricultural tools (hoe, machete), maize and bean seeds, and an unconditional cash payment of 263,000 UGS (~$100USD). Again, findings from this study indicate that an exclusive programme focus on abductees is problematic. We found no significant differences between former abductees and non-abductees in terms of survival/livelihood activities and experiences of food insufficiency, both overall and by gender. Similar proportions of both groups indicated that limited agricultural cultivation was their primary means of livelihood and that they earned a monthly income of less than 25,000 UGS, approximately $10USD (63% vs. 60%, *p =* 0.380). In addition, similarly high proportions of abductees and non-abductees had experienced and were currently experiencing food insufficiency. Food insufficiency was actually quite high overall (40% of respondents indicated a current lack of food and/or water, while 54% reported not having had enough food to eat in the previous 12 months). For nearly the past 15 years, the northern region of Uganda has been identified as one of the most food insecure regions in the country [31,32], and according to the 2011 Uganda AIDS Indicator Survey inhabitants of the North East (48%) and Mid Northern (47%) regions are disproportionately more likely to fall into the poorest wealth quintile while almost all residents of Kampala (95%) fall into the wealthiest quintile [33]. Additionally, men aged 15–49 residing in the Mid Northern region have the second lowest employment rate in the country, and men and women in the region are the least likely to be employed in the professional/technical/managerial and sales and services occupations in Uganda [33].

A lack of livelihood/poverty emerges as an overarching factor influencing the spread of HIV in conflict-affected environments, underpinning the sexual risks that women and girls take to obtain needed resources [29]. The principal dimensions of poverty during and after times of conflict include: the inadequate supply of basic essentials for the family, including food; the limited livelihood opportunities through which to gain these essentials, and; the breakdown of social and family structures, including the loss of the traditional role of men as the providers in the household. The struggle to survive within this environment where women face increased responsibilities for family upkeep compels women and girls to trade sex in exchange for food, water, shelter, protection and other basic commodities for themselves and their children [8,34-36]. Under these circumstances women are automatically vulnerable to potential financial and sexual exploitation by those who have access to food or money, most commonly men from their camps, camp committee members, traders, NGO workers, civil servants, and military and rebel soldiers [22,37]. The economic destitution of war-affected populations no doubt increases their HIV risk behaviours, and this is particularly the case for women and girls. Similarly, there is a growing body of literature that suggests that food insufficiency in times of conflict may enhance sex-related HIV vulnerabilities, particularly among females (i.e., survival sex, intergenerational sex, coerced sex) [38-41]. Mock et al. [2004] have argued that transactional sex in conflict settings is used as a coping strategy by women and girls who often have little or no access to resources [38]. Power dynamics come into play when women are food-dependent, enhancing the likelihood that condom use may not be negotiable. Furthermore, it is now generally accepted that those who are malnourished are more susceptible to HIV once exposure has occurred, as the lack of food weakens the immune system and compromises the mucosal membranes of the genital tract [42,43].

Our study finding, which, demonstrates no significant differences between former abductees and non-abductees in terms of survival/livelihood activities and experiences of food insufficiency, clearly show that the impacts of the war have been shouldered by everyone in northern Uganda, including all children and young people. It seems unfair and even counter-productive then that only formerly abducted persons are eligible for the Amnesty Commission’s re-insertion packages intended to help people rebuild their lives. This is not to say that young people who did not experience abduction are devoid of *any* assistance with re-establishing livelihoods, but rather, that aid agencies as well as government and NGO programmes may target formerly abducted persons disproportionately. Findings from the SWAY report (2007) suggest that the additional and exclusive assistance provided to former abductees continues to upset and offend many community members and that the unintended consequence of this approach to service delivery may be that former abductees are stigmatized rather than reintegrated [16]. It seems particularly unfair that the returnees who benefit from exclusive resources and services include former LRA commanders who committed gross violations of human rights against hundreds of thousands of civilians, while these civilians are seen as ‘mere victims’ and denied equal help. Having been at a reception centre or being eligible for an Amnesty Certificate are crude targeting measures that fail to identify the most vulnerable and underprivileged young people in northern Uganda. It is therefore imperative that programme planners and policy makers move away from a system of circumstantial categorization and instead develop responses for young people that target based on specific, easily identified, and acute needs. This would facilitate more effective post-conflict assistance and thereby strengthen the larger redevelopment process.

Based on a final finding from our study, one of those specific needs appears to be for post-conflict programmes offering support to victims of sexual violence to be inclusive of males and cognizant of their needs. Evidence on the sexual abuse of boys/men in conflict-affected populations is rare, but in this study 47% of sexually experienced male respondents (35% of all male participants) reported having been raped or sexually abused. Although the majority of research on sexual violence and HIV infection in times of conflict focuses on girls/women, there are many anecdotal accounts that suggest boys/men are no less susceptible to sexual violence than women during these times [44]. Closed environments such as IDP camps or rebels’ barracks are potentially conducive to male/male rape or other non-consensual sexual penetration [8]. Moreover, unprotected anal intercourse is a more effective means of HIV transmission than most other forms of sexual activity [45]. Eleven percent of the formerly abducted young men in this study reported that they were sexually abused in captivity. Sixteen percent of the sexually experienced male abductees reported a non-consensual sexual debut, 51% indicated that they had been abused by a sexual partner (34% in the previous 12 months), and 20% reported having been raped. Furthermore, male abductees were significantly more likely than male non-abductees to have experienced all of the above forms of sexual violence. Out of the 11 cases of reported rape among male abductees, 9 (82%) occurred at sexual debut. Four (44%) of the 9 male abductees who indicated that their first sexual experience was forced also reported that their first sex partner was 10 or more years older than they, and 7 (78%) reported that their first sex partner was a military/rebel soldier (who was most likely male, given the male dominance of military/rebel groups). In fact, 7 male participants in our sample overall indicated that the occupation of their first sex partner was a military/rebel soldier and all of these 7 young men were former abductees. The cross-sectional design of this study makes it difficult to determine with certainty that all experiences of sexual violence reported by formerly abducted young men in this study occurred while they were in captivity. This highlights the importance of conducting further research to advance an understanding of abducted males’ susceptibility to sexual violence during captivity. At the present time, however, our evidence strongly indicates that in northern Uganda, post-conflict programmes offering support to victims of sexual violence must recognize that males are also victims and thus must include them in programming that is sensitive to their needs and experiences.

This study has several limitations. First, attaining a probabilistic sample was a challenge. Our study results therefore only represent the young people we sampled and according to the principles of research methodology, cannot be generalized to all young people across northern Uganda. At the same time, it is important to note that over 90% of the population of northern Uganda was encamped during the war and that Gulu District was the most heavily impacted District in the region. Consequently, we believe that our sample population is characteristically similar to other conflict-affected young people in Gulu District and that our results *can* be generalized to this larger population. Second, the cross-sectional design of the study is a limitation because it did not allow us to determine if certain experiences known to elevate the risk of HIV (such as rape) occurred before, during or after abduction. Prospective studies that follow young people before and after abduction are required to make causal inferences in this regard. Third, this study relies on self-reported data, which may have been affected by the social desirability bias and recall errors. We aimed to minimize this affect by training our Acholi interviewers extensively, ensuring that all participants were interviewed by a research assistant of the same gender, and using a consent form that stressed the confidential and anonymous nature of the interviews – measures that have been shown to reduce response bias and maximize reliability among respondents [46]. Finally, analyzing abductees as a group without taking their length of time in captivity into account may have limited the implications of our study findings. That is, the specific programming needs that emerged in the study may not be applicable to all young people who have experienced abduction. However, we cautiously contend that the identified programming needs may be generalized to a certain extent. This is particularly so in the light of a large-scale survey that included 462 formerly abducted young northern Ugandans and demonstrated that extremely long-term abductions do not consistently predict poor well-being or specific vulnerabilities [16].

## Conclusions

This study persuasively demonstrates that *all* young people in northern Uganda are victims of war who continue to suffer and struggle due to the legacy of protracted conflict and displacement. Although the impacts of abduction are real and cannot be ignored, thousands of young people who have not been abducted are experiencing serious educational, economic, psychosocial, and health challenges. This indicates that abduction status may be a rudimentary and unreliable predictor of need. As programmes specifically targeted to former abductees exclude many vulnerable and underprivileged young people, they will ultimately undermine the larger redevelopment process. A one-size-fits-all approach to services for formerly abducted young people is likewise problematic because it does little to meet their varied needs. Furthermore, targeting programmes exclusively to former abductees could inadvertently cause them to be stigmatized and resented by communities.

This study supports recommendations that abduction status should qualify individuals for basic reinsertion support, but not be a special category, determinant, or precondition of aid [13]. Post-conflict programme planners and policy makers must abandon *adhoc* policies and rudimentary targeting practices based on abductees as a high-profile category. Instead, they must develop evidence-based HIV interventions that are commensurate with young people’s needs. Programmes that target based on specific and well-identified needs, such as the need for education, livelihood development, and support for victims of sexual violence, are likely to be more effective than programmes that target based on abduction status; to be more oriented to self-selection, and; to be less stigmatizing. The inclusive nature of these programmes will enable them to reach the most vulnerable young people in northern Uganda. As young people are a significant proportion of post-conflict society, they are vital to the recovery and reconstruction process. Indifference to them, however, does not bode well for the future of northern Uganda or the well-being of the next generation.

### Endnote

^a^In this paper, we use the term ‘child soldier’ interchangeably with the term ‘abductee’ to reflect the Paris Principles definition of children associated with armed forces and armed groups, which refers to “any child who is or who has been recruited or used by an armed force or armed group in any capacity, including but not limited to children, boys, and girls used as fighters, cooks, porters, messengers, spies, or for sexual purposes” [12]. It does not only refer to a child who is taking or has taken a direct part in hostilities.

## Competing interests

The authors declare that they have no competing interests.

## Authors’ contributions

SP conceived of and designed the study, drafted the manuscript, and performed the statistical analysis. MTS oversaw the analysis and interpretation of data and made critical revisions to the manuscript. SA and CO participated in the design and coordination of the study and made a substantial contribution to the acquisition of data. NK, PMS, and NKS provided guidance about the design of the study and the acquisition and interpretation of data. All authors read and approved the final manuscript.
